# †*Sorbinicharax verraesi*: An unexpected case of a benthic fish outside Acanthomorpha in the Upper Cretaceous of the Tethyan Sea

**DOI:** 10.1371/journal.pone.0183879

**Published:** 2017-08-28

**Authors:** Diogo Mayrinck, Paulo M. Brito, François J. Meunier, Jesus Alvarado-Ortega, Olga Otero

**Affiliations:** 1 Departamento de Ensino de Ciências e Biologia, Universidade do Estado do Rio de Janeiro, Rio de Janeiro, Brazil; 2 Departamento de Zoologia, Universidade do Estado do Rio de Janeiro, Rio de Janeiro, Brazil; 3 UMR 7208 (CNRS–IRD–MNHN –UPMC), BOREA, Département des Milieux et Peuplements Aquatiques, Muséum National d’Histoire Naturelle, CP026, Paris, France; 4 Departamento de Paleontología, Universidad Nacional Autónoma de México, Ciudad de México, México; 5 Laboratoire de Paléontologie de Poitiers – UMR 7262 Bat B35 – TSA51106, Poitiers, France; University of Michigan, UNITED STATES

## Abstract

†*Sorbinicharax verraesi* is a marine teleostean fish from the Upper Cretaceous of Nardò (Italy). It was first attributed to the otophysan order Characiformes, which represents potential evidence for the controversial marine origin of the clade. Through a review of all the available material, we demonstrate that this species is not an otophysan since it lacks key structures that would allow for its inclusion in this group. †*Sorbinicharax* has a body shape that recalls ground fishes classically assigned to Acanthomorpha. However, no unambiguous feature allows us to relate it to this clade. In fact, the presence of cellular bony tissue supports its exclusion from Eurypterygii. Since no feature permits the definitive attribution of †*Sorbinicharax* to any teleost group, it remains as Teleostei *incertae sedis*. We infer that the morphology of †*Sorbinicharax* indicates a benthic ecology. It displays: an anteriorly wide body with enlarged ribs; large pectoral fins, while anal and dorsal fins are reduced; a large head measuring ¼ of the total body length; and a mouth opening dorsally in a high position. Such morphology was so far undescribed in Nardo. It is surprisingly displayed by a non-eurypterygian teleost fish which means by a fish which does not belong to the clades that diversify since the upper Cretaceous and include the extant families that show ground ecomorphologies.

## Introduction

Since the 1980’s, several controversial fossils have heated the debates about the primary environment for the early diversification of otophysan fishes [1–7]. †*Sorbinicharax verraesi* Taverne, 2003a occupied a peculiar position since it was the youngest described and the only one attributed to the Characiformes, a group known by its freshwater ecology [8]. †*Sorbinicharax verraesi* was described from Upper Cretaceous deposits of Nardò, a marine Tethyan locality in Southern Italy. According to Taverne [9], †*Sorbinicharax* is an otophysan fish, based on a well-developed Weberian apparatus, the absence of basisphenoid and the fusion of the second hypural and the compound terminal centrum in the caudal skeleton. Taverne [9] included †*Sorbinicharax* in a new family (†Sorbinicharacidae) and placed it as a primitive marine member of the Characiformes based on certain shared apomorphies proposed by Fink & Fink [1, 2] for the order.

We recently started an anatomical and systematic review of marine fossil fishes attributed to Otophysi by one or several authors. So far, we have found that †*Chanoides macropoma* (Middle Eocene, Monte Bolca) and †*Nardonoides chardoni* (Coniacian-Campanian, Nardò, Italy) are effectively stem-group otophysan fishes, that “†*C*. *weberi*” is nomen nudum and that †*Salminops ibericus* is not an otophysan but rather is related to crossognathiforms [7, 10].

This paper focuses on †*Sorbinicharax verraesi* and is based on the anatomical review of the only two specimens discovered so far. On this basis, we discuss the systematic position of the species and its morphological convergence with certain modern ground fishes.

## Materials and methods

### Materials

The only materials are two specimens from the marine deposits of Nardò, Southern Italy, dated as Campanian-Maastrichtian (see [11] for details on datation). The holotype MCSNV Na 499 is almost complete, with the exception of the caudal skeleton, which is preserved as imprints. The other exemplar is preserved as part and counterpart and numbered MCSNV Na 47 (right) and MCSNV Na 48 (left, almost imprints). These paratypes reveal complementary details of the first vertebrae, the otic bulla, part of the neurocranium, part of the hyoid bar, and the pectoral girdle and fin. The material is deposited in the Museo Civico di Storia Naturale di Verona, Italy. No permits were required to conduct this study, which complied with all relevant regulations and national law of Italy.

The observations were made with a binocular microscope, and the photographs were taken under low-angle light using a Sony α57 digital camera.

### Methods

#### Ground sections

A fragment of the skull roof belonging to the exemplar MCSNV Na 48 was removed, embedded in stratyl resin and sectioned for histological examination. The sections were polished down to a thickness of approximately 80 μm and observed under transmitted natural and polarized light with a Nikon Eclipse E66 POL and with a Zeiss Axiovert 35.

#### Institutional abbreviations

MCSNV, Museo Civico di Storia Naturale di Verona, Italy.

## Systematic paleontology

Teleostei Müller, 1846 [12]

Teleostei *incertae sedis*

Family †Sorbinicharacidae Taverne, 2003 [9]

Genus †*Sorbinicharax* Taverne, 2003 [9]

### Revised diagnosis

Small fish of approximately 15 mm in standard length and 18 mm in total length; head equal to about one third of the standard length; supraorbital sensory canal in a tube on the frontals; small mesethmoid; basisphenoid process present; unicuspid jaw teeth; edentulous maxilla; premaxilla with a short ascending process; dentary with probably a single row of teeth; articulation between the quadrate and maxilla posterior to the orbit level; extrascapular not fused to the parietals; well-developed otic bulla; losangic metapterygoid and quadrate-metapterygoid fenestra; interopercle with a large horizontal extension to the front of the ventral surface of the quadrate; high hyomandibula; pharyngeal bones with a pair of stout toothpatches with unicuspidate, globular teeth; pharyngeal teeth larger than mandibular ones; 26 vertebrae, 11 abdominal and 15 caudal; first vertebral centrum foreshortened; second vertebral centrum longer than the others; three anteriormost pairs of ribs enlarged inserted on centra 3 to 5; parapophyses developed on the last four abdominal vertebrae; dorsal fin with at least 9 rays placed above the posterior part of the abdominal cavity as well as the pelvic fin; small anal fin with at least 4 rays; peripheral ctenoid scales.

### Type species

†*Sorbinicharax verraesi* Taverne, 2003 [9] from the Coniacian-Campanian of Nardò, Southern Italy.

### Holotype

MCSNV Na 499.

### Paratypes

MCSNV Na 47, Na 48 (part and counterpart).

### Remark

Some diagnostic characters of Taverne [9] are not emended because they are impossible to observe. In fact, the structures concerned are either covered by scales, impossible to observe or not preserved. These are: the opening of the temporal fossa and certain features of the first three infraorbitals; the antorbital and supra-orbital; the hemapophyses of the first vertebra; counts and patterns of fusion in the caudal skeleton, and the absence of a rhinosphenoid and of a supra-preopercle.

### Species diagnosis

As for the genus.

## Results

### Anatomical description

**Fig 1:**
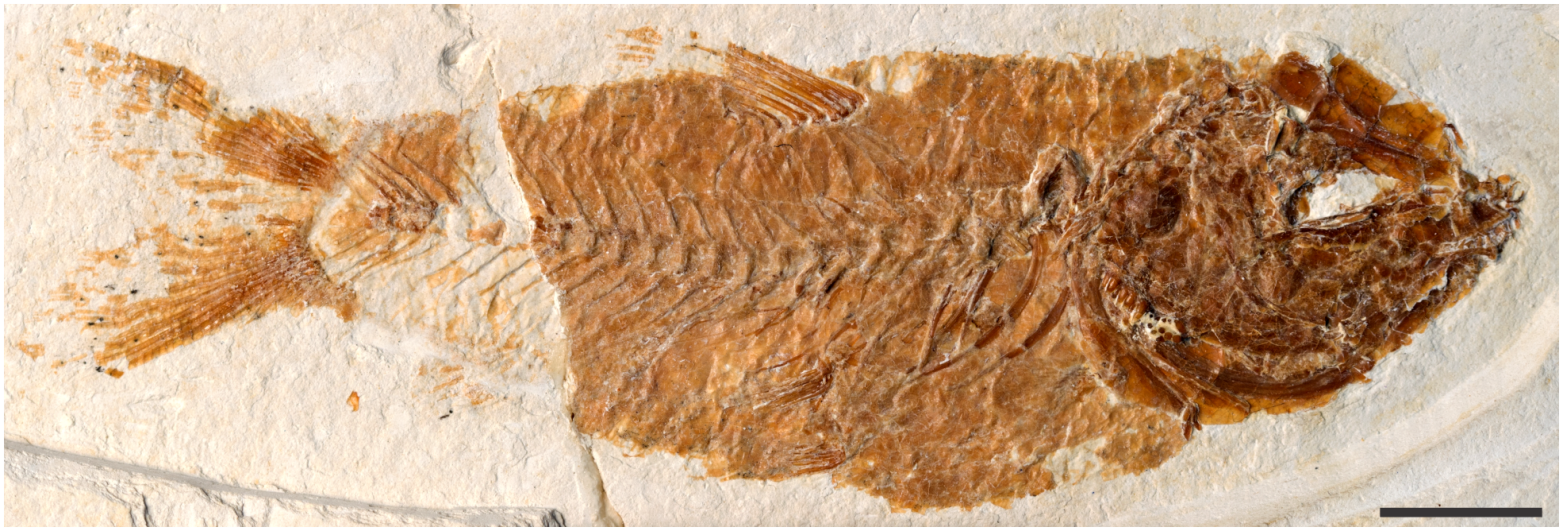
Photo of holotype of †*Sorbinicharax verraesi*. Exemplar MCSNV Na 499. Scale bar = 5 mm.

†*Sorbinicharax verraesi* is a small fish of less than 2 cm in total length (Fig 1). The body is wide anteriorly, with a large head (about a quarter of the total length) that ends with a small prognathous mouth. According to Taverne [9], the body is covered by ctenoid scales. More precisely, they correspond to the peripheral ctenoid type as defined by Roberts [13], i.e., with the ctenii seen as spines aligned in one row at the scale margin.

#### The head

The bones of the head are poorly preserved on both specimens, and when present they are often crushed so that their limits are not defined. The mesethmoid is badly preserved and mostly covered by the posterodorsal process of the premaxilla, the shape and exact position of which are hard to distinguish. The mesethmoid possibly inserts between the anterior ends of the frontals. The vomer is only preserved in its anteriormost region, where a few teeth are present, in contrast to Taverne’s [9] which described an edentulous, large and long bone.

**Fig 2:**
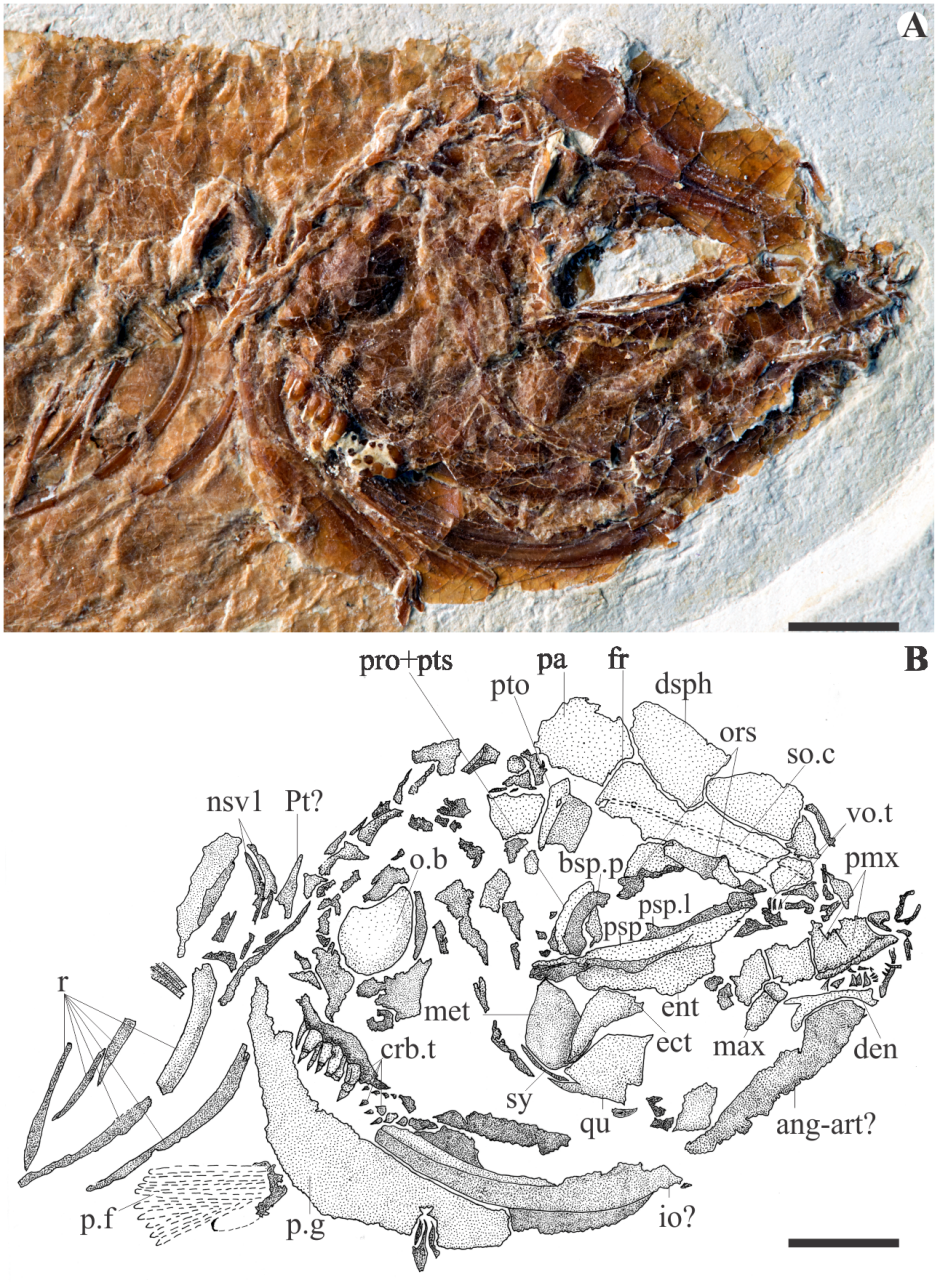
Details of the head of the exemplar MCSNV Na 499. **A**, photograph; **B**, interpretive drawing. Abbreviations: ang-art, angulo-articular; bsp.p, basisphenoid process; crb.t, ceratobranchial teeth; den, dentary; dsph, dermosphenotic; ect, ectopterygoid; ent, endopterygoid; fr, frontal; io, interopercle; max, maxilla; met, metapterygoid; nav1, neural arch of the vertebral centrum 1; o.b, optic bulla; ors, orbitosphenoid; p.f, pectoral fin; p.g, pectoral girdle; pa, parietal; mx, premaxilla; pro, prootic; psp, parasphenoid; psp.l, lateral wing of the parasphenoid; pt, post-temporal; pto, pterotic; pts, pterosphenoid; qu, quadrate; r, ribs; so.c, supraorbital sensory canal; sy, sympletic; vo.t, vomerian teeth. Scale bar = 5 mm.

**Fig 3:**
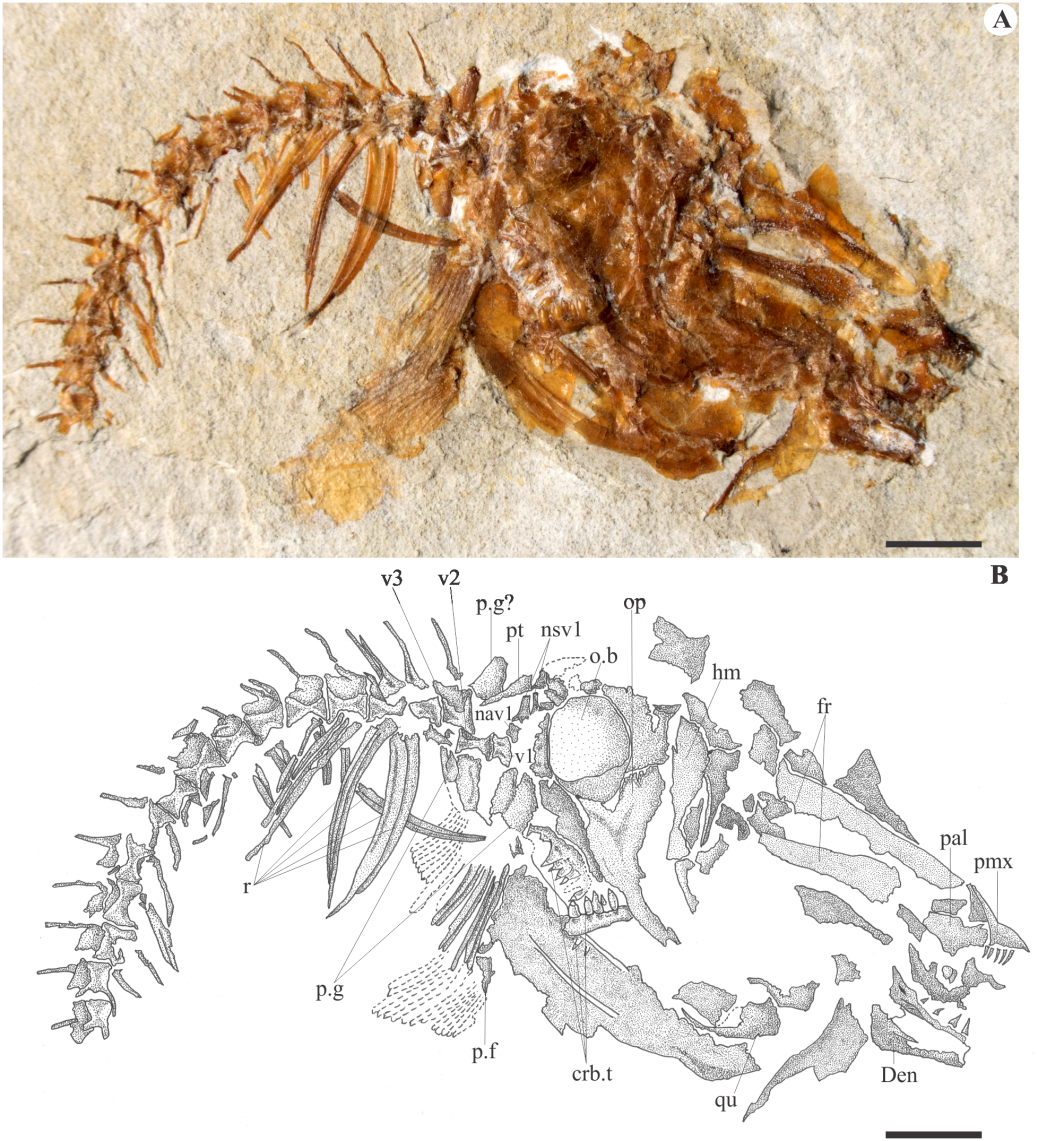
Paratype of †*Sorbinicharax verraesi*. Exemplar MCSNV Na 47. **A**, photograph; **B**, interpretive drawing. Abbreviations: crb.t, ceratobranchial teeth; den, dentary; fr, frontal; hm, hyomandibular; nav1, neural arch of the vertebral centrum 1; nsv1, neural spine of the vertebral centrum 1; o.b, optic bulla; op, opercle; p.f, pectoral fin; p.g, pectoral girdle; pal, palatine; pmx, premaxilla; pt, post-temporal; qu, quadrate; r, ribs; v, vertebral centrum. Scale bar = 5 mm.

The frontals display a bony tube running from the anterior to the posterior edge for the passage of the supraorbital sensory canal. The parietals contact the frontals anteriorly in a straight, transversally oriented, suture line. On MCSNV Na 499 (Fig 2), the large parietal is also seen sutured anteriorly to the frontal and anterolaterally to the dermosphenotic or to the dermopterotic. The pterotic is preserved displaced with its posterior projection oriented ventrally on MCSNV Na 47 (Fig 3). Contrary to Taverne’s (2003a) description, the epiotic and the supraoccipital are lacking, and there is no evidence of a post-temporal fossa or of any extrascapular commissure.

The prootic is preserved as a bony mass in the anteromedial area of the otic region. A basisphenoid process sutures dorsally to the prootic and ventrally to the parasphenoid. In both MCSNV Na 499 and MCSNV Na 47, a marked bulla occupies the posterior edge of the head, and is covered by the opercle (Figs 2 and 3). Its position within the otic region and the inner ear is hard to ascertain, and we found no argument to interpret it more precisely, including as a lagenar bulla as proposed by Taverne [9]. The orbitosphenoid projects anteriorly toward the ethmoidal region in a large process, seen in the specimen MCSNV Na 499 (Fig 2). According to Taverne [9], the parasphenoid is long and edentulous, and neither basipterygoid nor ascending processes are seen. On MCSNV Na 499, the median portion of the parasphenoid exhibits a lateral wing below the orbit (Fig 2).

The upper jaw is formed by a stout toothed premaxilla with a marked ascending process and a probably small and toothless maxilla that lacks articulation with the mesethmoid (Figs 2 and 3). In the lower jaw, the well-developed anguloarticular bone is twice as large as the dentary, which bears a single row of teeth. The retroarticular and the articulation of the lower jaw with the quadrate are not preserved. Jaw teeth are conical and equal in size and shape (Figs 2 and 3). The hyomandibular is well preserved in MCSNV Na 47 (Fig 3). It exhibits a large dorsal edge with two articular condyles: an anterior one with the autosphenotic and a posterior one that articulates with the pterotic. A straight horizontal shaft of the hyomandibular is also partially preserved. The rest of the cheek is better preserved in MCSNV Na 499 (Fig 2). The lozenge-shaped metapterygoid has a rounded ventral tip that contacts the posterodorsal portion of the quadrate. *Versus* Taverne [9], there is no metapterygoid-quadrate fenestra (which implies an X-shaped metapterygoid). The ectopterygoid is curved and lies onto the anterolateral surface of the quadrate. The endopterygoid is roughly oval in shape.

**Fig 4:**
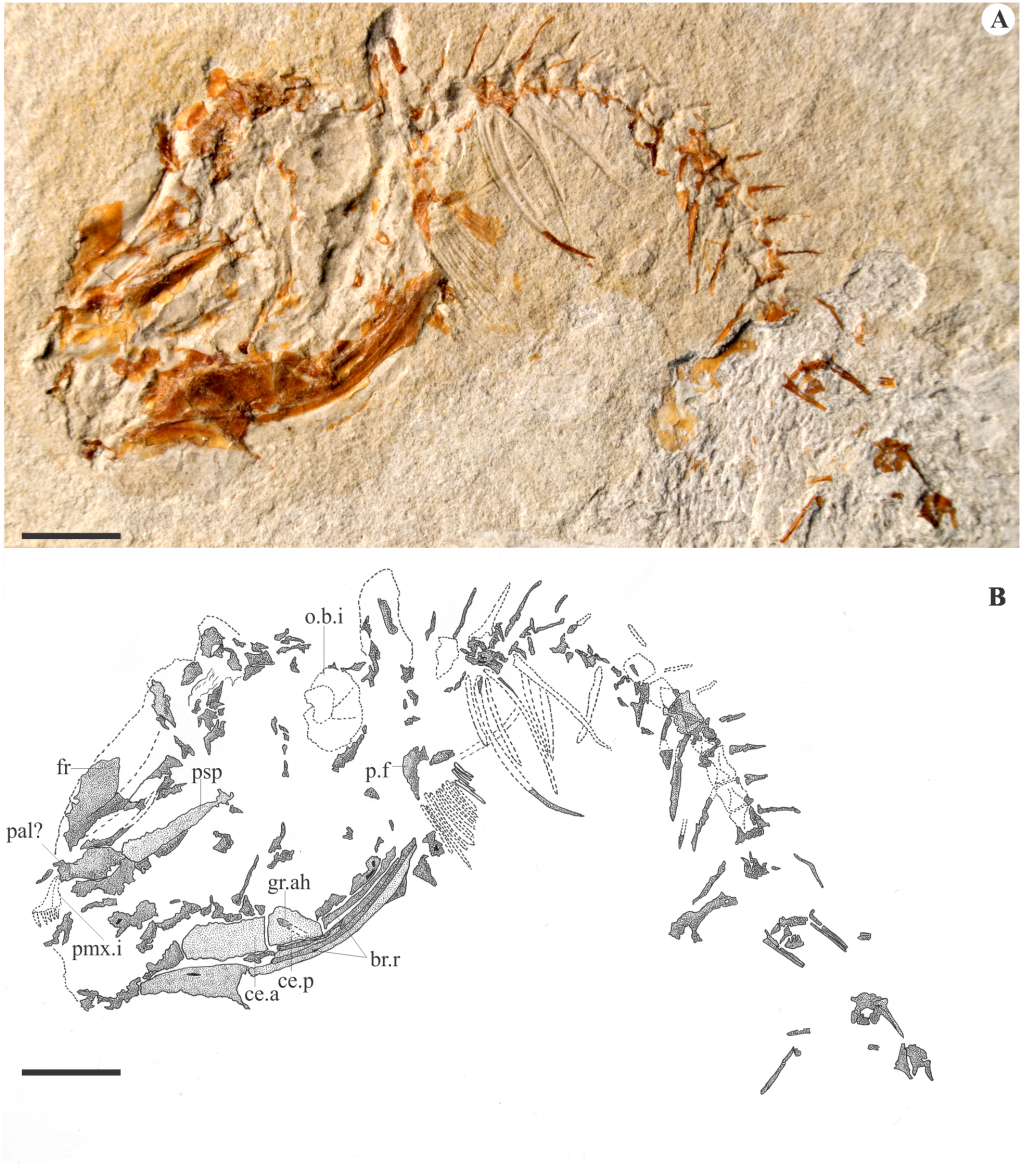
Paratype of †*Sorbinicharax verraesi*. Exemplar MCSNV Na 48. A, photograph; B, interpretive drawing. Abbreviations: br.r, branchiostegal ray; ce.a, anterior ceratohyal; ce.p, posterior ceratohyal; crb.t, ceratobranchial teeth; fr, frontal; gr.ah, groove for the hyoid artery; o.b.i, optic bulla imprint; p.f, pectoral fin; pal, palatine; pmx.i, premaxilla imprint. Scale bar = 5 mm.

The opercular series includes a significantly well-developed bone, probably the interopercle, that extends frontwards below the quadrate. The branchial skeleton is partially known from well-toothed pharyngeal jaws. A dorsal toothplate is probably associated with the posteriormost pharyngobranchials, while a ventral one is probably associated with the fifth ceratobranchials. The teeth are more than twice as large as the mandibular ones and are arranged in at least two rows on each plate (Figs 2 and 3). Seen on MCSNV Na 48 (Fig 4), the anterior ceratohyal is almost rectangular in shape, while the posterior ceratohyal is oval shaped with a deep groove for the hyoid artery extending along the anterodorsal edge onto the lateral surface. A series of massive branchiostegal rays is seen partially preserved in the exemplar MCSNV Na 48. It includes at least 5 rays that are twice larger than the hyoid bar.

#### The axial skeleton

Information on the vertebral column is available on both MCSNV NA 499 and Na 47 (Figs 1 and 3). However, the anteriormost part is covered in the former specimen and, in the latter, is found slightly displaced (the first and second vertebrae are displaced ventrally) and covered by bony fragments that probably belong to ribs and to the pectoral girdle. There seem to be 26 vertebrae, among which 11 are abdominal, with parapophyses on the last four ones. Neural spines are seen from the third vertebra backwards, and that of at least the fifth centrum is bifid. All are short and directed backwards in the abdominal region when compared to the post-abdominal region. At least the three anteriormost pairs of ribs are stout, long and enlarged. *Versus* Taverne [9], there is no trace of any modification of the anteriormost vertebrae and associated bones, and thus, no structure that might be interpreted as a Weberian apparatus. The dorsal fin presents at least nine rays that are articulated serially with pterygiophores. The fin is aligned with and above the pelvic fins and the posterior part of the abdominal region. The anal fin is poorly preserved and includes at least four rays.

**Fig 5:**
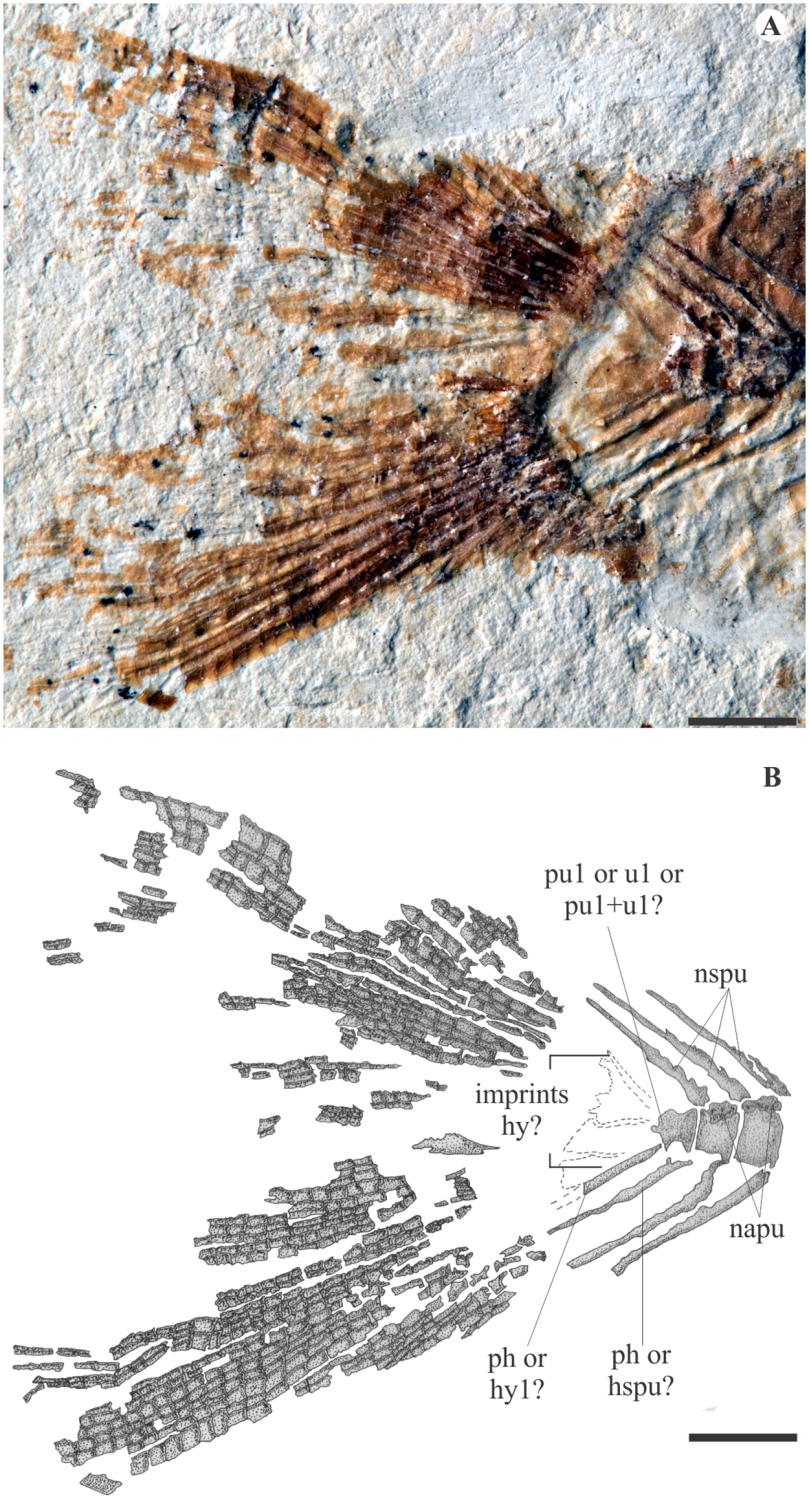
Details of the caudal skeleton of †*Sorbinicharax verraesi*. Exemplar MCSNV Na 499. **A**, photograph; **B**, interpretive drawing. Abbreviations: hspu, hemal spine of the preural centrum; hy, hypural; napu, neural arch of the preural centrum; nspu, neural spine of the preural centrum; ph, parhypural; pu, preural centrum; u, ural centrum. Scale bar = 5 mm.

The caudal skeleton is poorly preserved, and the details described by Taverne (2003a) are not available except the forked outline of the fin (Fig 5). Three preural centra with their related neural arch and fused neural spines are seen, all well developed. Posteriorly, it is not possible to further identify the axial elements. The hypurals, epurals and uroneurals are preserved as irregular imprints, and their shape and number cannot be determined. Taverne [9] described nineteen principal rays, of which seventeen branched. This cannot be excluded, though a lower count of eight dorsal plus nine ventral seems more appropriate to us.

#### The pair fins and their skeleton

The pectoral girdles and fins are mostly preserved on MCSNV Na 47 (Fig 3). There is a massive and large cleithrum. The coracoid and scapula are too poorly preserved on both specimens to be precisely delimited. The supracleithrum and the posttemporal are partially preserved. The former is displaced beneath the second centrum on MCSNV Na 499 (Fig 2). According to Taverne [9], there are at least eighteen pectoral rays, which cannot be confirmed since the fin is preserved as an imprint with almost imperceptible ray boundaries. We count at least preserved twelve rays, possibly up to 16. At least some anterior rays are branched.

The pelvic girdles are preserved in MCSNV Na 499. They are located below the dorsal fin level at mid-length of the abdominal cavity. They are covered by scales except in the ischium region, which shows a lateral projection and bears at least twelve rays; the first one is bifurcated proximally (Fig 1).

#### Histology

**Fig 6:**
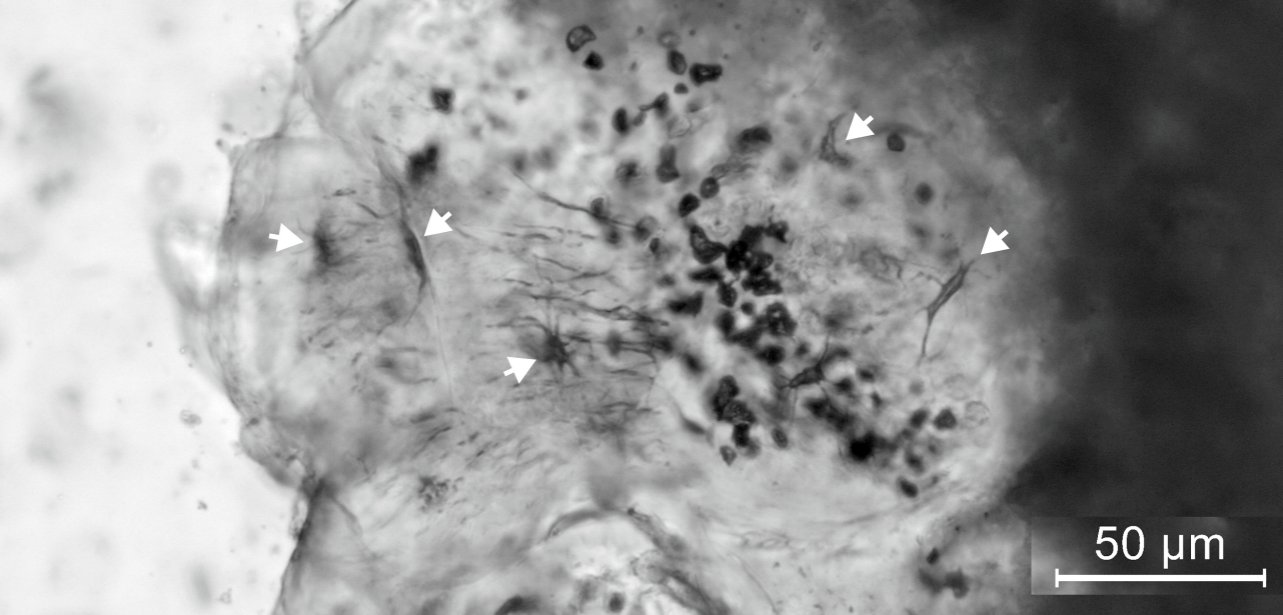
Ground section of fragments of the skull roof of †*Sorbinicharax verraesi*. Exemplar MCSNV Na 48. The white arrows indicate the star-shaped cells and their cytoplasmic extensions. Scale bar in μm (micrometers).

On a thin section of a fragment of the skull roof, histological details of the bone of †*Sorbinicharax verraesi* are observed. The bone is cellular, with star-shaped cells and well-marked cytoplasmatic extensions (Fig 6).

## Discussion

### On the systematic status of †*Sorbinicharax verraesi*

†*Sorbinicharax verraesi* was described as a characiform by Taverne [9] based on the presence of several synapomorphies for the order proposed by Fink & Fink [1, 2]. However, none of these features can be found on the fossil material because the relevant bones or structures are either lacking, misinterpreted or over interpreted: (1) the relative position of the vomer and the mesethmoid and the articulation of the premaxilla and the maxilla with the mesethmoid remain unknown as the mesethmoid cannot be located; (2) if a large otic bulla is seen, no element permits identification it as lagenar bulla; (3) the presence of a mediodorsal opening of the posttemporal fossa between the epiotic and pterotic is impossible to ascertain; (4) due to the lozenge shape of the metapterygoid, no metapterygoid-quadrate fenestra can be present as seen in the MCSNV Na 499, which is better preserved in that region (Taverne [9] identified this structure on MCSNV Na 47, which is completely crushed there); (5) the anteriormost part of the axial skeleton was described with details that fit with a Weberian apparatus, and more particularly with a characiform one (or supposed as such, in the case of the scaphium, which is not a paired element as presented in the original description, but an unpaired element); but indeed, the vertebrae are not modified at all, and the so-called “Weberian ossicles” are fragments of the top of the pelvic girdle and ribs, lying slightly displaced onto the anteriormost vertebrae; (6) Taverne [9] described a characiform-like caudal skeleton, but most of it, notably the key structures such as the compound terminal centrum, cannot be detailed.

Therefore, concerning its former assignment to a characiform fish, †*Sorbinicharax* is now completely excluded from this order due to the lack of any Weberian structures and of any compound terminal centrum in the caudal skeleton.

Certain anatomical features, including a low number of abdominal vertebrae and the presence of peripherical ctenoid scales, are common in acanthomorph fishes. Therefore, one attractive hypothesis would have been to suggest a placement within acanthomorphs, which were largely diversified during the Late Cretaceous. However, the presence of none of the osteological apomorphies of this clade can be observed [14, 15, 16, 17, 18]. In addition, the presence of cellular bone in *†Sorbinicharax* (Fig 6) prevents the attribution to Acanthomorpha since all of them are known to have acellular bone with the exception of a very small number of endothermic species that exhibit secondary cellular bone [19; pers. obs.]. The presence of cellular bone in *†Sorbinicharax* (Fig 6) also prevents relationship with non acanthomorph Eurypterygii since the Aulopiformes and Myctophiformes are documented to have acellular bones [19, 20], which also suggests that acellular bone is a symplesiomorphic state for Ctenosquamata and possibly for Eurypterygii [21]. Since *†Sorbinicharax* is not a eurypterygian fish, the pharyngobranchial jaw dentition probably developed onto the fifth elements and the supraorbital bone was possibly absent [22, 23]. Finally, we did not find any unambiguous apomorphy specifically relating this Cretaceous fish to any specific teleost clade. This is mainly due to the poor preservation of the specimens, as mentioned throughout the paper, and possibly to its derived morphology (see below). Therefore, †*Sorbinicharax verraesi* is neither an otophysan nor an acanthomorph (and more generally a eurypterygian fish) and should be considered as a Teleostei *incertae sedis*.

### About the probable ecology of †*Sorbinicharax verraesi*

**Fig 7:**
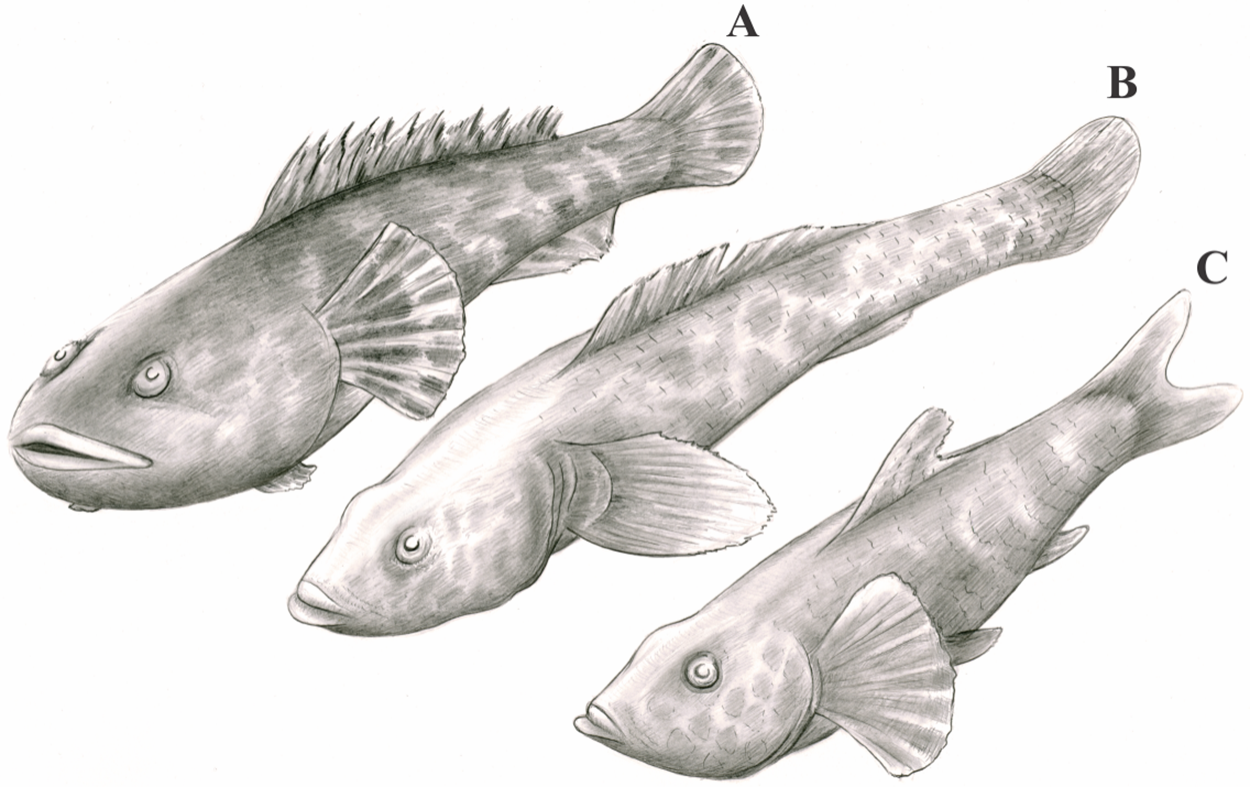
Artistic reconstruction of two extant benthic fish and of †*Sorbinicharax verraesi*. A, toadfish (*Batrachoides sp*.); B, goby (*Neogobius sp*.); C, †*Sorbinicharax verraesi*. To reconstruct †*Sorbinicharax verraesi* shape we followed the midline of the holotype body interpreted from the position of the dorsal and pelvic fins. For the shape of the mouth and pectoral fin we also include complementary information from the paratypes specimens.

†*Sorbinicharax verraesi* exhibits a morphology and some anatomical features that converge, at least partially, with several extant rocky ground benthic fishes, notably gobies and toadfishes (Fig 7): it has a massive head relative to its body length (one quarter of the total length); the body itself is wider than it is tall, which explains why remains of the back scales are preserved on both sides of the dorsal fin as well as ventral skin on both sides of the pelvic skeleton; the anteriormost ribs are large; the pelvic and pectoral fins are well developed, and the wide, rounded pectoral fins are at mid-height on the body; the dorsal fin is placed above the posterior half of the abdominal cavity, and both dorsal and anal fins are relatively short; the large head is characterized by the high position of the small mouth that might have opened dorsally and with stout arches. Finally, another striking feature is the remarkably stout dentition on the pharyngeal jaws, which contrasts with the small pointed teeth at the premaxillae, vomer and dentaries. In these main lines, the dentition of †*Sorbinicharax verraesi* resembles certain cichlids or centrarchids for instance. These fish have a relatively flexible diet, ranging from snails and bivalves to omnivory [24]. Notwithstanding, the authors noted also a variable response of their teeth to their diet, from more delicate and pointed morphologies to crushing dentition. However, other features that are not evaluated here, such as the crushing strength, are also determinant to allow a shell-diet [25].

However, two other features commonly found in benthic fishes are lacking: a rounded caudal fin and a long-based dorsal fin (†*S*. *verraesi* exhibits a forked tail and a short-based dorsal fin). Indeed, we suspect that a long-based dorsal fin, although characteristic of most benthic fish families, is not always found associated with this habitat. For instance, gobiesocids may have a rather short-based dorsal fin. Conversely, forked-tailed benthic fish are scarce amongst acanthomorphs but less in aulopiforms. Indeed, in benthic fish the posterior edge of the caudal fin commonly exhibits a convex outline with a large range of shapes: mainly from rounded to straight, but the caudal fin may also exhibit a spear head shape or may end the body in a simple spike. These features evidently depend on a balance on both ecology and phylogeny. Thus, lacking any information on a precise systematic position for †*Sorbinicharax*, it is impossible to discuss further whether the lack of a convex outline of the posterior edge and of a long-based dorsal fin alone invalidate our hypothesis of a benthic habit for the taxon. So, lacking any other evidence to further define its ecology, we assume that the body anatomy, the jaws morphology and the dentition of †*Sorbinicharax verraesi* correspond to benthic fish feeding mainly on small animals, possibly catching the prey as an ambush predator by a combination of suction and protrusion of the jaws [26, 27].

### Why was †*Sorbinicharax verraesi* twice unexpected at Nardò?

The environment of the Melissano limestone corresponds to a carbonate platform with peritidal facies and rudist bivalves [28], and we easily can imagine a demersal fish community feeding on bivalves and on other benthic organisms. However, so far only pelagic species were identified in this locality, in which acanthomorphs are the most diverse single in terms of named species [14, 29, 30, 31]. More generally, we were not able to find any Cretaceous fish with such a “benthic adapt morphology” with the exception a few macrosemiid fishes [32, 33]. In that respect, †*Sorbinicharax verraesi* was unexpected in the Melissano levels. Its presence considerably extends the fish morphological diversity at Nardò and also questioned about the conditions that favored the emergence of benthic morphologies trough paleontological times.

Today, most of the marine fish diversity and the morphological disparity are due to acanthomorph fishes [18]. They notably include most of the marine benthic fishes with the notable exceptions of elopomorph fishes that display anguilliform morphologies and of certain aulopiform fishes such as lizardfish and flagfins. However, most of their morphological diversification appears to be consequence of recovery in the aftermath of the Cretaceous-Paleogene extinction, followed by successive waves of innovation during the Paleogene [31, 34]. For instance, the earliest modern reef fish communities appear later, in the Eocene, notably in another Italian locality, at Monte Bolca [34, 35, 36, 37]. Therefore, because acanthomorph and aulopiform fish diversify since the Cenomanian (as far as seen in the fossil record), we would have rather expected that upper Cretaceous tentatives to adapt demersal waters with such a morphology would have been promoted by one of their member. However, †*Sorbinicharax verraesi* does probably not belong to Acanthomorpha or Aulopiformes as discussed above. In that sense, it documents a so far unknown failed tentative to conquer benthic habitat by a non eurypterygian and non-otophysan fish during the Upper Cretaceous.

## Conclusion

†*Sorbinicharax verraesi* had a benthic ecology, and in this sense, it increases the ecological fish diversity hosted in Nardò’s marine shallow waters approximately 80 million years ago. Moreover, it presents a case of a benthic stocky morphology among marine teleosts, unique in its timing and by the group concerned. It occurs before and outside the acanthomorph adaptive radiation to coastal and reef environments that began in the Upper Cretaceous but only occurred during the Tertiary for benthic fish. This early experimentation of a benthic morphology might be related with the pre-reef environments of the Melissano limestone near Nardò. It probably became extinct as this morphology and anatomy were so far unknown.

Beside these eco-morphological issues, we suspect that the derived morphology of †*Sorbinicharax verraesi* reduces our chances to attribute it more precisely. The presence of cellular bone certainly excludes it from Eurypterygii and moreover prevents its placement in any family that today exhibits this type of morphology. It seems to belong to an extinct lineage in another marine teleost fish clade, the descendants of which today exhibit different morphology. The benthic adaptation may have hidden a few features that would have been informative for placing †*Sorbinicharax verraesi*, notably on the axial and fin skeletons. Finally, its morphology also probably played a role in the poor preservation of the head and thus acts to prevent any phylogenetic placement of this fish. However, it remains surprising how few fish displayed a ground-fish ecomorphology during the Upper Cretaceous at a time when reef environments occupies the extendly submerged continental shelves produced by the dislocation of the Pangea.
